# High-Conductivity Solid-State Electrolytes Through Low-Temperature Hot-Pressing of LCBA/LATP Composites

**DOI:** 10.3390/ma19102033

**Published:** 2026-05-13

**Authors:** Wookyung Lee, Jaeseung Choi, Jungkeun Ahn, Hanbyul Lee, Byungwook Kim, Youngsoo Seo, Changbun Yoon

**Affiliations:** 1 Department of Advanced Materials Engineering, Tech University of Korea, Siheung-si 15073, Gyeonggi-do, Republic of Korea; ds090390@tukorea.ac.kr (W.L.); jaejae0303@tukorea.ac.kr (J.C.); quddnr000@tukorea.ac.kr (B.K.);; 2 Battery R&D Team, Samsung SDI, 150-20, Gongse-ro, Giheung-gu, Yongin-si 17084, Gyeonggi-do, Republic of Korea; woodtrains@naver.com

**Keywords:** all-solid-state battery, lithium battery, solid electrolyte, LCBA glass, LATP, low-temperature, composite

## Abstract

**Highlights:**

- Low-temperature (600 °C) hot-pressed LCBA/LATP composite electrolyte fabricated.
- 3:7 composition achieved 2.40 g/cm^3^ density and 2.5 × 10^−4^ S/cm conductivity.
- Uniform phase stability and effective densification suggested by XRD/SEM.
- Enables co-sintering with graphite and Si anodes below 600 °C.
- Improved Li interfacial stability compared with pristine LCBA or LATP.
- Promising low-temperature route for scalable ASSB manufacturing.

**Abstract:**

Solid-state electrolytes (SSEs) are essential for achieving long-term stability and fast-charging performance in secondary batteries. Although Li_1.3_Al_0.3_Ti_1.7_(PO_4_)_3_ (LATP) offers high ionic conductivity, its practical application is restricted by high-temperature sintering requirements and interfacial reduction at the lithium anode. In contrast, Li-based oxide electrolytes can be sintered below 600 °C, offering improved compatibility with conventional electrodes such as graphite and silicon. In this study, a Li_2_O–LiCl–B_2_O_3_–Al_2_O_3_ (LCBA)/LATP composite SSE was fabricated via hot-press co-sintering at 600 °C. Composites with LCBA:LATP weight ratios of 8:2, 7:3, 6:4, 5:5, 3:7, and 2:8 were prepared to identify the optimal composition. The 3:7 composite achieved a sintered density of 2.40 g/cm^3^ and an ionic conductivity of 2.5 × 10^−4^ S/cm. Phase evolution and sintering behavior were characterized by X-ray diffraction (XRD) and scanning electron microscopy (SEM). Compared to single-phase LCBA or LATP, the composite electrolyte exhibited improved interfacial stability and lower interfacial resistance against lithium metal.

## 1. Introduction

Secondary batteries are essential for modern technology, powering applications ranging from portable electronics and electric vehicles to uninterruptible power supplies and grid-scale energy storage [1,2,3]. Lithium-ion batteries (LIBs) currently dominate this sector due to their high energy density, favorable charge–discharge efficiency, and long life cycle [4,5,6]. However, conventional LIBs rely on organic liquid electrolytes, which pose significant safety risks, including electrolyte decomposition, gas generation, lithium dendrite growth, short-circuiting, and thermal runaway [7,8,9,10,11]. Beyond safety concerns, the energy density of liquid electrolyte-based LIBs is approaching its practical limit under current electrode chemistries, necessitating the development of next-generation battery technologies [12,13,14,15,16].

All-solid-state batteries (ASSBs) have emerged as a promising alternative by replacing liquid electrolytes with solid electrolytes (SEs). This substitution offers several practical benefits, including reduced dendrite penetration risk, improved thermal stability, longer cycle life, and higher theoretical energy density [17,18,19,20,21,22]. Research on ASSBs primarily focuses on two SE families: sulfide-based [18,23,24,25] and oxide-based [26,27,28,29,30] materials.

Sulfide-based SEs exhibit high ionic conductivity, typically on the order of 10^−2^ S cm^−1^ and approaching that of liquid electrolytes [31,32]. Nevertheless, their reactivity to atmospheric moisture, which can lead to the release of toxic hydrogen sulfide gas, presents significant processing and safety challenges [24,33,34,35,36].

In contrast, oxide-based SEs are chemically stable under ambient conditions [37,38,39,40,41,42,43,44,45,46]. Several of these have shown room-temperature ionic conductivities above 1 mS cm^−1^. Their primary drawback is the requirement for high-temperature sintering, typically exceeding 1000 °C, which can increase interfacial resistance at the electrode–electrolyte interface and degrade electrode materials [47,48,49]. Specifically, carbon-based additives and graphite anodes begin to oxidize above 600 °C [50,51,52].

To address this limitation, glass-based SEs have been investigated as a lower-temperature alternative for ASSBs [53,54,55,56]. Lithium-ion-conducting glasses and glass ceramics sinter below 600 °C, improving compatibility with conventional electrode materials. However, their primary limitation is relatively low ionic conductivity, with values on the order of 10^−5^–10^−6^ S/cm [52,54]. While thermal treatment above the glass transition temperature (T_g_) can induce partial crystallization to enhance ionic transport, maintaining a balance between processability and conductivity remains challenging [52,57].

Despite these advances, a practical gap remains; no single SE system simultaneously satisfies the requirements of low sintering temperature, high ionic conductivity, and stable electrode interfaces. Glass-based SEs address the temperature constraint but lack sufficient conductivity, whereas high-conductivity oxides such as LATP require processing conditions that can lead to incompatibility with standard electrode materials and interfacial instability against lithium metal. Composite approaches have been proposed to combine these advantages; however, systematic experimental data on composition-dependent densification and electrochemical behavior in such hybrid systems remain limited.

In this study, a glass-LATP composite (GLC) SE consisting of Li_2_O–LiCl–B_2_O_3_–Al_2_O_3_ (LCBA) glass and Li_1.3_Al_0.3_Ti_1.7_(PO_4_)_3_ (LATP) was fabricated for multilayer ceramic battery (MLCB) applications. Using hot-press sintering at 600 °C, composites with LCBA:LATP weight ratios of 8:2, 7:3, 6:4, 5:5, 3:7, and 2:8 were prepared and their sintered density and ionic conductivity were evaluated.

The composite exhibited improved ionic conductivity compared to the individual constituents. The 3:7 LCBA/LATP composition achieved a sintered density of 2.40 g/cm^3^ and an ionic conductivity of 2.5 × 10^−4^ S/cm. Given that LATP is susceptible to reduction at the anode interface and LCBA may react with cathode materials, the composite configuration may help to mitigate these limitations, although further interface characterization is required to confirm this behavior [58].

## 2. Materials and Methods

### 2.1. Fabrication of LCBA and LATP Composites

To synthesize LATP, the following high-purity reagents were used: Li_2_CO_3_ (99%, Sigma-Aldrich Korea, Seoul, Republic of Korea), Al_2_O_3_ (99%, Sigma-Aldrich Korea, Seoul, Republic of Korea), TiO_2_ (99%, Sigma-Aldrich Korea, Seoul, Republic of Korea), and NH_4_H_2_PO_4_ (99%, Sigma-Aldrich Korea, Seoul, Republic of Korea). The LATP powder was synthesized via a conventional wet solid-state reaction. Stoichiometric amounts of the starting materials, corresponding to the composition of Li_1.3_Al_0.3_Ti_1.7_(PO_4_)_3_, were weighed and mixed by ball milling at 150 rpm for 4 h in alcohol. The resulting slurry was transferred to an alumina crucible and calcined at 850 °C for 2 h to form the LATP phase. The calcined powder was subsequently re-milled at 150 rpm for 24 h and sieved to obtain powder of controlled particle size.

LCBA glass powder was prepared via a conventional solid-state reaction involving powder mixing and ball milling, using precursor materials sourced from Daejoo Electronic Materials (Siheung-si, Gyeonggi-do, Republic of Korea). Thermal analysis indicated a glass transition temperature (T_g_) of 423 °C and a crystallization temperature (T_c_) of 487 °C, confirming the material’s suitability for heat treatment below 600 °C. LCBA and LATP powders were combined in weight ratios of 8:2, 7:3, 6:4, 5:5, 3:7, and 2:8 and then mixed by ball milling at 150 rpm for 2 h using zirconia balls.

The mixed powders were uniaxially pressed into green pellets (15 mm diameter, 1.0 mm thickness) at 110 MPa. Hot-press sintering was performed under uniaxial pressure during heat treatment. For comparison, pressureless sintering was also performed by placing green pellets in an alumina crucible and heat-treating for 3 h under otherwise identical conditions. In both cases, a heating rate of 5 °C/min was maintained, and the sintering temperature was held at 600 °C for 60 min. Following sintering, the pellet surfaces were ground with abrasive paper to remove surface contaminants, and the final thickness was adjusted to 0.5 mm.

### 2.2. Battery Performance Evaluation of LCBA/LATP Composite Solid Electrolytes

The crystal structure of the composite SEs was characterized by XRD (D2 PHASER, Bruker, Billerica, MA, USA). Cross-sectional morphology and the interfacial regions between the composite and lithium metal were examined by scanning electron microscopy (Nova NanoSEM 450, FEI, Hillsboro, OR, USA). Additionally, the spatial distribution of P and Ti within the LCBA matrix was mapped using energy-dispersive X-ray spectroscopy (EDS, NanoSEM, FEI, Hillsboro, OR, USA) to evaluate the dispersion of LATP particles.

Electrochemical impedance spectroscopy (EIS) and linear sweep voltammetry (LSV) were performed using an impedance analyzer (VersaSTAT 3, Princeton Applied Research, Oak Ridge, TN, USA) with VersaStudio software (version 2.67.3, AMETEK, Inc., Berwyn, PA, USA). For electrochemical characterization, an Au/LCBA–LATP composite/Au cell was assembled by depositing gold electrodes on both sides of the sintered composite pellet, and EIS was performed over a frequency range of 0.1 Hz–100 kHz with an AC amplitude of 5 mV. Additionally, an Au/LCBA–LATP composite/Li cell was assembled by depositing a gold electrode on one side of the sintered composite pellet while lithium metal was placed on the opposite side, and electrochemical stability was examined by LSV over a voltage range of 2.5–5.5 V at a scan rate of 1 mV/s.

Interfacial stability was evaluated using CR2032-type coin cells, where sintered LCBA/LATP pellets were sandwiched between two lithium metal electrodes. Galvanostatic cycling was carried out at a current density of 0.001 mA/cm^2^ using a battery testing system (WBCS3000S, WonATech, Seoul, Republic of Korea) operated with Smart Interface software (version 1.8.9.0, WonATech Co., Ltd., Seoul, Republic of Korea).

## 3. Results and Discussion

### 3.1. Thermal Stability and Crystalline Structural Analysis

**Figure 1:**
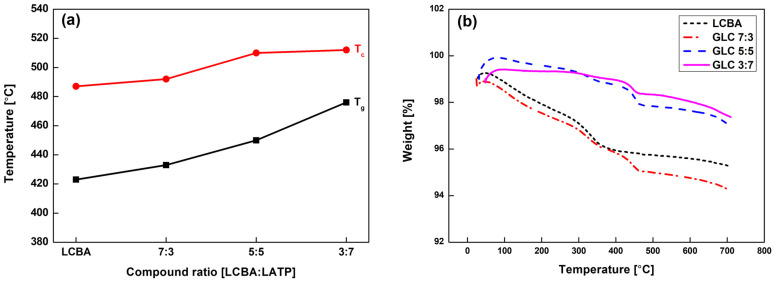
Thermal stability and phase transition characteristics of GLCs: (**a**) thermal transitions and (**b**) TGA curves.

The thermal stability and phase transition characteristics of LCBA and GLC composites with ratios of 7:3, 5:5, and 3:7 are shown in Figure 1. As presented in Figure 1a, both the glass transition temperature (T_g_) and crystallization temperature (T_c_) are shown as a function of LATP content. For pure LCBA, T_g_ and T_c_ were observed at 423 °C and 487 °C, respectively. In the GLC 7:3 composition, both values increased slightly to 433 °C and 492 °C. As the LATP content increased further, this trend continued: T_g_ and T_c_ reached 450 °C and 510 °C for GLC 5:5 and 476 °C and approximately 512 °C for GLC 3:7. The monotonic increase in both transition temperatures with increasing LATP content indicates that LATP incorporation raises the crystallization onset of the composite.

TGA results (Figure 1b) are consistent with this trend. Pure LCBA and GLC 7:3 showed continuous mass loss from around 100 °C, while composites with higher LATP content exhibited a more gradual decrease. At 700 °C, the residual weight increased with LATP content, with GLC 3:7 and GLC 5:5 retaining approximately 97% of their initial mass, compared to roughly 94% for LCBA and GLC 7:3. This suggests that a higher LATP fraction contributes to greater thermal mass retention across the measured temperature range.

**Figure 2:**
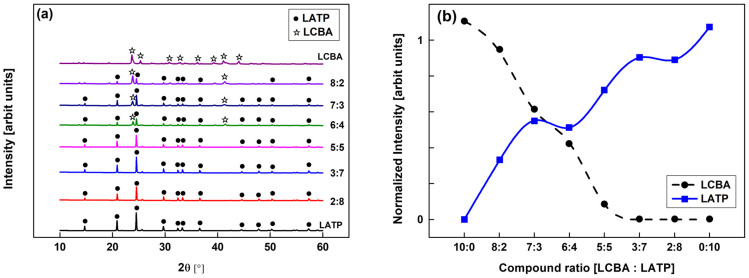
Crystal structure and peak intensity analysis of LATP and LCBA composites: (**a**) X-ray diffraction patterns and (**b**) normalized intensity.

XRD analysis was conducted to examine the structural characteristics of the GLC composites as a function of the LCBA:LATP mixing ratio. As shown in Figure 2a, diffraction peaks corresponding to both LATP and LCBA phases were identified across all compositions, with no additional peaks attributable to secondary phases or reaction products. Within the detection limits of XRD, these results suggest that the two constituents coexist without evident chemical interaction under the applied processing conditions.

The normalized peak intensities of the primary diffraction peaks for each phase in Figure 2b show that as the LATP fraction increases from 0 to 100 wt%, the LATP peak intensity increases progressively while that of LCBA decreases correspondingly. Both trends show a degree of nonlinearity near the 7:3 composition, which may reflect changes in phase distribution or local microstructural arrangement.

At the 3:7 (LCBA:LATP) composition, the diffraction peaks of both phases remain clearly distinguishable. The retention of well-defined peaks from each constituent at this ratio suggests that neither phase undergoes significant structural disruption upon mixing, and that both are present as discrete crystalline domains within the composite.

### 3.2. Sintering Behavior and Microstructural Densification

**Figure 3:**
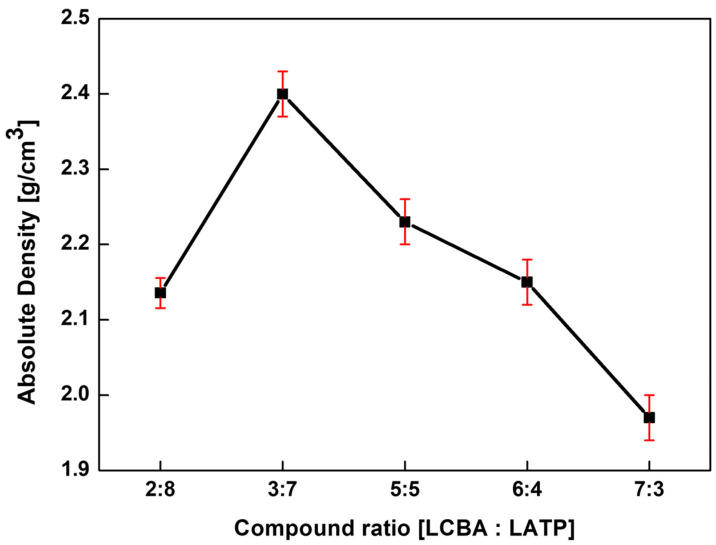
Absolute density of GLCs as a function of the compound ratio.

The absolute density of the GLC composites exhibits a bell-shaped trend as a function of composition (Figure 3), rather than following a simple weighted average of the constituent densities.

The density increased from approximately 2.13 g/cm^3^ at a ratio of 2:8 to a maximum of approximately 2.40 g/cm^3^ at the 3:7 composition. This increase may be related to differences in particle size between the two phases, where finer particles of one component occupy interstitial voids between coarser particles of the other, resulting in improved packing during sintering. At the 3:7 ratio, particle rearrangement during sintering appears to be most effective, resulting in a relatively dense microstructure with reduced internal porosity.

Beyond the 3:7 composition, density decreased progressively with increasing LCBA content, falling to approximately 2.23, 2.15 and 1.97 g/cm^3^ for the 5:5, 6:4, and 7:3 compositions, respectively. Two factors may contribute to this trend. First, higher LCBA fractions may promote particle agglomeration, leading to non-uniform mixing and localized shrinkage during sintering that increases closed porosity. Second, as the LCBA—which has a lower intrinsic density than LATP—becomes dominant, the overall composite density decreases accordingly.

Taken together, these density data indicate that the 3:7 (LCBA:LATP) composition ratio yields the highest sintered density among the compositions evaluated. The relatively small standard deviation observed across repeated measurements suggests reasonable reproducibility within this compositional range.

**Figure 4:**
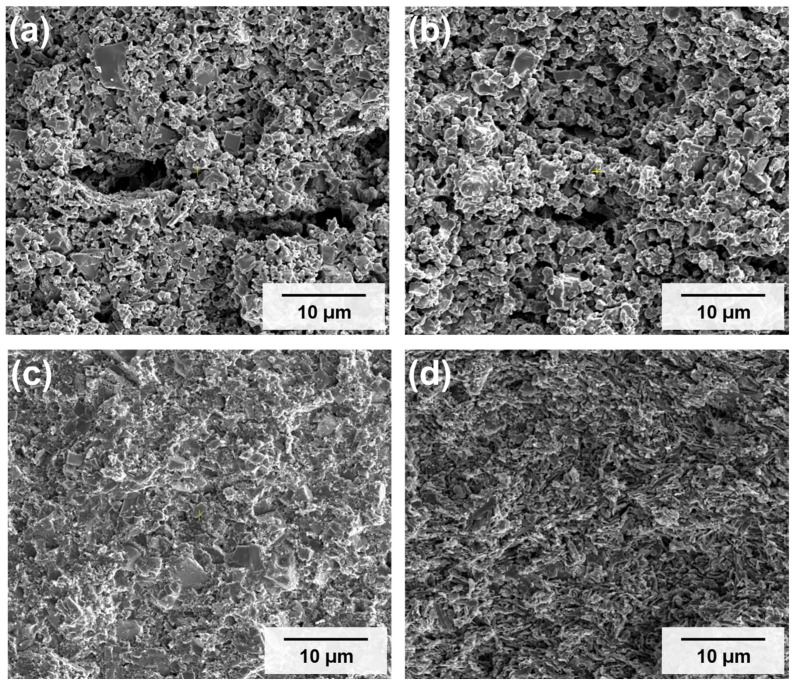
SEM images of GLCs sintered at 600 °C, comparing samples with and without applied pressure. Cross-sectional images of press-less pellets: (**a**) 5:5 and (**b**) 3:7. Cross-sectional images of hot-pressed pellets: (**c**) 5:5 and (**d**) 3:7.

Figure 4 illustrates the effects of sintering pressure and composite ratio on the cross-sectional microstructure of GLC compacts sintered at 600 °C. Press-less samples (Figure 4a,b) exhibited a considerable number of irregular macropores across both 5:5 and 3:7 compositions. This porosity may be associated with limited atomic diffusion between particles under press-less conditions, resulting in incomplete inter-particle bonding.

In contrast, the hot-pressed samples (Figure 4c,d) showed a notable difference in microstructural characteristics relative to their press-less counterparts. The application of uniaxial pressure during sintering reduced interstitial voids, resulting in reduced porosity and a denser microstructure with improved inter-particle contact. Among the hot-pressed samples, the 3:7 composite (Figure 4d) exhibited a more compact and uniform cross-sectional morphology compared with the 5:5 sample (Figure 4c), which appears to reflect more uniform particle distribution at this mixing ratio.

The SEM observations are broadly consistent with the absolute density data discussed previously. The comparison between press-less and hot-pressed samples indicates that external pressure during sintering is associated with reduced porosity and continuous inter-particle contact, particularly at the 3:7 composite ratio. Quantitative porosity estimates are summarized in Table 1.

**Figure 5:**
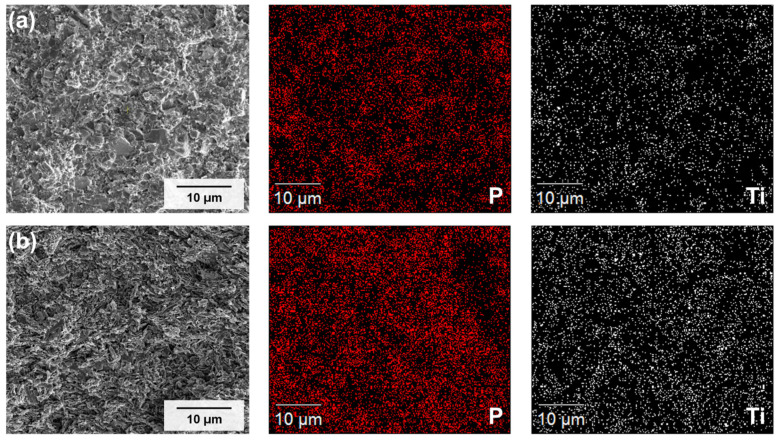
SEM images and corresponding EDS elemental mapping (P and Ti) of the hot-pressed GLCs: (**a**) 5:5 and (**b**) 3:7 ratios.

Figure 5 presents SEM images and corresponding EDS elemental maps for P and Ti in hot-pressed GLC composites with LCBA:LATP ratios of 5:5 (Figure 5a) and 3:7 (Figure 5b).

In both compositions, P and Ti were uniformly distributed across the mapped region, with no evidence of localized aggregation or elemental segregation. Phase separation between the two constituents was not observed under the applied sintering conditions. This spatial distribution of both elements is consistent with the formation of a mixed composite structure in which the LCBA and LATP phases are intermixed at the microscopic level.

A comparison between the two compositions indicates that the 3:7 sample (Figure 5b) shows a slightly denser and more uniform distribution of elemental signal relative to the 5:5 sample (Figure 5a). This difference may be associated with reduced inter-particle voids in the 3:7 composition, which may increase the effective signal density per unit area in EDS mapping. Overall, the EDS results suggest that hot-pressing produced a compositionally uniform microstructure in both samples, with the 3:7 composition showing greater consistency in elemental distribution.

### 3.3. Electrochemical Analysis of Ionic Conductivity

**Figure 6:**
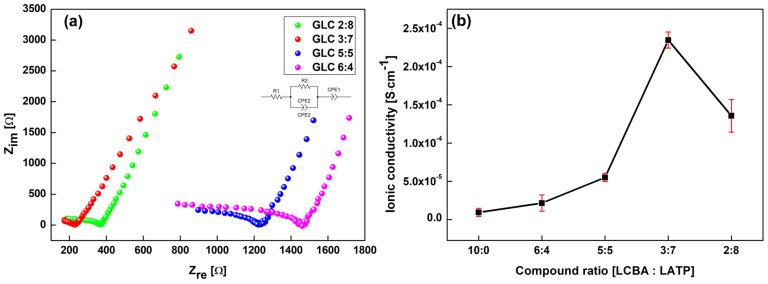
Ionic conductivity analysis of GLCs heat-treated at 600 °C with various composition ratios: (**a**) Nyquist plots of GLCs with different mixing ratios (6:4, 5:5, 3:7, and 2:8); (**b**) ionic conductivity as a function of the ratios of GLCs.

EIS measurements were performed on GLC composites heat-treated at 600 °C to examine the variation in ionic conductivity as a function of the LCBA:LATP mixing ratio (Figure 6).

The Nyquist plots in Figure 6a show that the semicircle diameter in the high-frequency region, which represents the combined bulk and grain boundary resistances, varied with composition. The GLC 3:7 sample exhibited the smallest semicircle and the lowest intercept on the real axis (Z*_re_*), corresponding to the lowest internal resistance among all compositions tested. Samples with higher LCBA content, including 6:4 and 5:5, exhibited larger semicircle diameters, indicating higher internal impedance.

The ionic conductivity (σ) values derived from the impedance data (Figure 6b) follow a non-linear trend as a function of composition. The ionic conductivity was calculated from the total resistance obtained by equivalent circuit fitting using

(1) $$\sigma =\frac{t}{R_{total}\times A}$$

where *t* is the pellet thickness, *A* is the electrode contact area, and *R_total_* is the sum of bulk and grain boundary resistances. Starting from a low value at the LCBA-rich end, conductivity increased with increasing LATP fraction, reaching a maximum of approximately 2.5 × 10^−4^ S/cm at the 3:7 composition. Among the compositions evaluated, this ratio yielded the highest ionic conductivity.

Beyond the 3:7 composition, the ionic conductivity decreased at the 2:8 ratio. The reason for this decline is not immediately apparent from the data alone and may warrant further investigation. The error bars at each data point in Figure 6b are relatively small, suggesting reasonable reproducibility across measurements.

**Figure 7:**
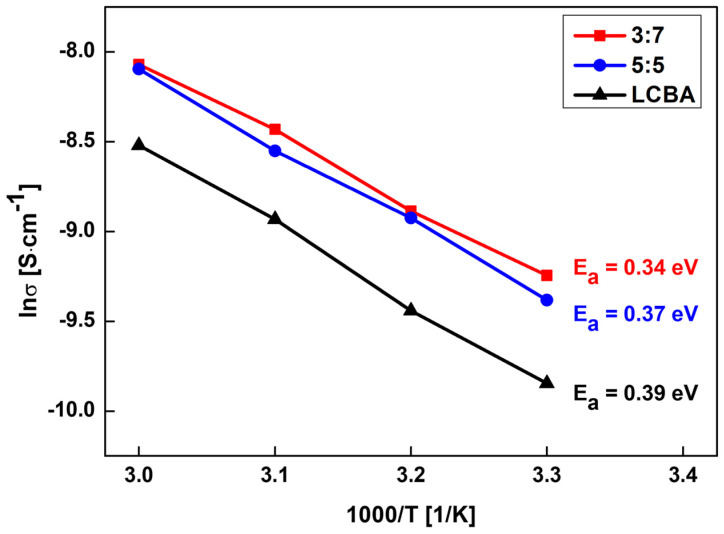
Arrhenius plot of pure LCBA and GLC composites (5:5 and 3:7), indicating temperature-dependent ionic conductivity and the calculated activation energy.

Figure 7 shows the Arrhenius plot of lnσ versus 1000/T for pure LCBA and GLC composites with 5:5 and 3:7 ratios, measured over the temperature range corresponding to 1000/T = 3.0–3.4 K^−1^.

All three samples exhibited a linear relationship between lnσ and 1000/T across the measured temperature range, with high coefficients of determination. This linearity indicates that ion transport in these materials follows a thermally activated process with a single dominant mechanism and that no apparent phase transitions or structural changes occurred within the measured temperature range.

The activation energy (E_a_) was determined from the slope of each linear fit using the Arrhenius equation. The calculated values were 0.39 eV for pure LCBA, 0.37 eV for the 5:5 composite, and 0.34 eV for the 3:7 composite. E_a_ represents the energy barrier that lithium ions must overcome during migration between sites within the solid framework. The decrease in E_a_ from LCBA to GLC 5:5 to GLC 3:7 indicates a progressive reduction in this barrier with increasing LATP content, which may be related to the microstructural densification observed at the 3:7 composition.

The 3:7 composite showed both the highest ionic conductivity and the lowest E_a_ among the three samples, and the linear Arrhenius behavior was maintained across the full measured temperature range.

**Figure 8:**
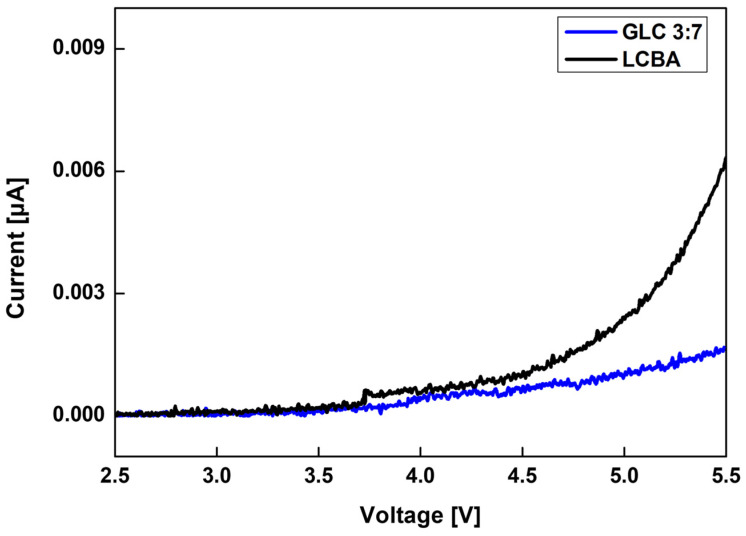
LSV profiles of pure LCBA and GLC 3:7 composite.

Figure 8 presents the LSV profiles of pure LCBA and the GLC 3:7 composite, measured over a voltage range of 2.5–5.5 V at a scan rate of 1 mV/s. The measurements were conducted using an Au/composite/Li cell configuration to evaluate the electrochemical response of each material under applied voltage.

For pure LCBA, a gradual increase in current was observed across the measured voltage range, with the rate of increase becoming more pronounced at higher voltages. In contrast, the GLC 3:7 composite maintained a comparatively moderate current response throughout the same voltage range, with lower overall current values relative to pure LCBA. This difference suggests that the incorporation of LATP into the composite contributes to a degree of delay in current increase within the measured voltage window.

Both samples showed current values approaching zero in the 2.5–3.5 V range, indicating that no pronounced oxidative reactions were detected for either material within this region. Across the full measured voltage range, the GLC 3:7 composite exhibited a more restrained increase in current, particularly at higher voltages. These results indicate that the composite configuration is associated with a somewhat delayed onset of electrochemical reaction relative to pure LCBA and are consistent with stable behavior over the voltage range relevant to secondary battery applications.

**Figure 9:**
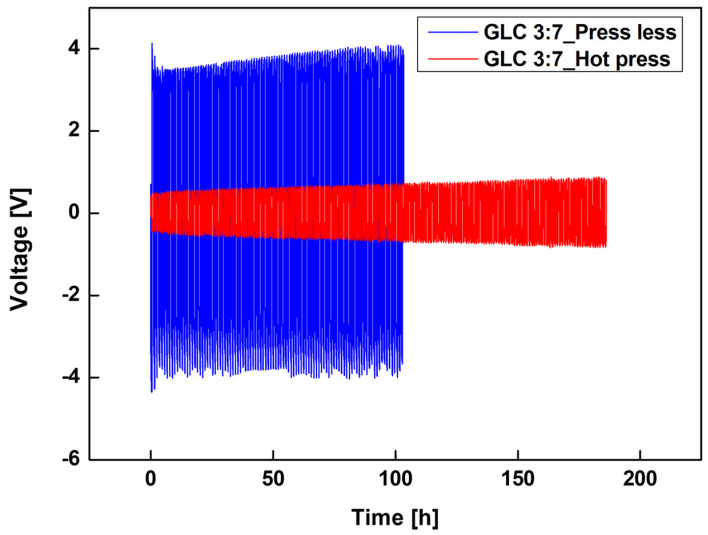
Long-term electrochemical stability of 3:7 GLC in Li–Li symmetric cells: evolution of overpotential and cycling stability, comparing the effects of hot-pressing and press-less sintering.

Figure 9 shows the galvanostatic cycling performance of Li–Li symmetric cells assembled with GLC 3:7 electrolytes sintered under two different conditions to evaluate the effect of the sintering process on interfacial behavior over time.

The press-less sample (GLC 3:7_Press less) exhibited unstable cycling behavior from the outset, with an overpotential of approximately 4 V throughout the initial cycling period. This elevated overpotential may reflect high porosity and limited electrode–electrolyte contact. At approximately the 100 h mark, the voltage profile collapsed abruptly toward 0 V, suggesting the occurrence of an internal short circuit.

In contrast, the hot-pressed sample (GLC 3:7_Hot press) maintained a low and stable overpotential within approximately ±0.5 V for over 190 h. The voltage profile remained symmetrical and flat throughout cycling, with no notable increase in overpotential during extended operation. This behavior is consistent with more stable electrode–electrolyte contact in the hot-pressed sample compared to the press-less counterpart.

Taken together, the cycling data indicate that the sintering condition had a measurable effect on the electrochemical stability of the GLC 3:7 electrolyte in Li–Li symmetric cells, with the hot-pressed sample showing more stable behavior over the duration of the test. A comparison of representative oxide-based solid electrolytes and their electrochemical properties is summarized in Table 2.

## 4. Conclusions

This study examined the effects of the LCBA:LATP composite ratio and sintering conditions on the physical and electrochemical properties of GLC solid electrolytes. XRD analysis (Figure 2) confirmed that the characteristic diffraction peaks of both LATP and LCBA were identified across all mixing ratios, with no evidence of secondary phase formation or chemical interaction between the two constituents. The normalized peak intensities showed a composition-dependent trend with some deviation from linearity, and at the 3:7 ratio, both phases remained distinguishable with no apparent structural disruption.

Microstructural analysis indicated that densification depends on the application of pressure during sintering. Press-less samples exhibited considerable macroporosity with limited inter-particle contact, whereas hot-pressed samples showed reduced porosity and improved inter-particle contact. Among the evaluated compositions, the hot-pressed 3:7 sample yielded the highest absolute density of 2.40 g/cm^3^. EDS elemental mapping (Figure 5) was consistent with these observations, showing a uniform distribution of P and Ti in both the 5:5 and 3:7 samples with no evidence of elemental segregation, consistent with a well-mixed composite structure.

Electrochemical characterization showed that structural densification was associated with changes in ionic conduction performance. The 3:7 composition achieved the highest ionic conductivity of approximately 2.5 × 10^−4^ S/cm among the ratios evaluated (Figure 6). Arrhenius analysis (Figure 7) yielded the lowest activation energy of 0.34 eV for this sample, indicating relatively low barriers to ion migration over the measured temperature range. LSV measurements further indicated a more restrained current response in the GLC 3:7 composite relative to pure LCBA across the measured voltage range (Figure 8). Li–Li symmetric cell testing (Figure 9) showed that the hot-pressed 3:7 GLC electrolyte maintained a stable voltage profile for over 190 h, with no evidence of short-circuit over that period, whereas the press-less sample showed voltage instability and apparent short-circuit behavior at approximately 100 h. Taken together, these results suggest that the combination of hot-pressing and a 3:7 composite ratio provides optimal microstructural and electrochemical performance in GLC solid electrolytes.

## Figures and Tables

**Table 1:** Porosity and densification parameters of LCBA and GLC composite electrolytes.

Sample	Relative Density (%)	Porosity (%)
Press-less GLC 5:5	86.4 ± 1.2	13.6
Press-less GLC 3:7	89.1 ± 0.8	10.9
Hot-pressed GLC 5:5	94.7 ± 0.5	5.3
Hot-pressed GLC 3:7	97.8 ± 0.3	2.2

**Table 2:** Comparison of representative oxide-based solid electrolytes and their electrochemical properties.

Oxide-Based SE	Structure	Sintering Temp. (°C)	Ionic Conductivity [S/cm]	Characteristic	Ref.
LLZO	Garnet-type	1100~1200	10^−4^~10^−3^	High σ; poor Li wettability; co-sintering incompatible	[28,44,45,46]
LATP	NASICON	900~1100	10^−4^~10^−3^	High σ; Ti^4+^ reduction at Li anode; co-sintering incompatible	[39,40,41]
LCBA	Oxide glass	400~600	10^−6^~10^−5^	Low sintering temp.; low σ	[52,54]
[This work] GLC	Glass-ceramic	600	2.5 × 10^−4^	Low sintering temp.; improved σ; stable Li interface (>190 h)	-

## Data Availability

The original contributions presented in the study are included in the article, further inquiries can be directed to the corresponding author.

## Abbreviations

The following abbreviations are used in this manuscript:

LIBs	Lithium-ion batteries
ASSBs	All-solid-state batteries
SEs	Solid electrolytes
MLCB	Multilayer ceramic battery
GLC	Glass–LATP Composite
